# Iatrogenic cerebral arterial gas embolism from flushing of the arterial line in two calves

**DOI:** 10.1186/s13028-018-0405-5

**Published:** 2018-09-06

**Authors:** Daniela Casoni, Alessandro Mirra, Christine Goepfert, Ilaria Petruccione, Claudia Spadavecchia

**Affiliations:** 1 Department of Clinical Veterinary Sciences, Vetsuisse Faculty, University of Berne, Längassstrasse 124, 3012 Berne, Switzerland; 2 Department of Infectious Diseases and Pathobiology, Vetsuisse Faculty, University of Berne, Länggassstrasse 122, 3012 Berne, Switzerland

**Keywords:** Calf, Cerebral infarcts, DIC, Gas embolism, Invasive blood pressure

## Abstract

**Background:**

Measurement of invasive blood pressure as reflection of blood flow and tissue perfusion is often carried out in animals during general anesthesia. Intravascular cannulation offers the potential for gas to directly enter the circulation and lead to arterial gas embolism. Cerebral arterial gas embolism may cause a spectrum of adverse effects ranging from very mild symptoms to severe neurological injury and death. Although several experimental models of arterial gas embolism have been published, there are no known published reports of accidental iatrogenic cerebral arterial gas embolism from flushing of an arterial line in animals.

**Case presentation:**

A 7-day-old Red Holstein–Friesian calf (No. 1) and a 28-day-old Holstein–Friesian calf (No. 2) underwent hot iron disbudding and sham disbudding, respectively, under sedation and cornual nerve anesthesia. Invasive arterial blood pressure was measured throughout the procedure and at regular intervals during the day. Before disbudding, a sudden and severe increase of blood pressure was observed following flushing of the arterial line. Excitation, hyperextension of the limbs and rapid severe horizontal nystagmus appeared shortly thereafter. Over the following minutes, symptoms ameliorated and blood pressure normalized in both cases. Prompt diagnosis was missed in calf 1; supportive fluid therapy was provided. Severe deterioration of neurologic status occurred in the following 24 h and culminated with stupor. The calf was euthanized for ethical reasons and the histological examination revealed extensive cerebral injury. Treatment of calf 2 consisted of supportive fluid and oxygen therapy; furosemide (1 mg/kg IV) was injected twice. Calf 2 appeared clinically normal after 2 h and showed no neurologic sequelae on a 3-month-follow up period.

**Conclusions:**

There are no known reports of cerebral arterial gas embolism following flushing of the auricular arterial line in calves. The injection of a small amount of air at high pressure in a peripheral artery may lead to a significant cerebral insult. The clinical presentation is non-specific and can favour misdiagnosis and delay of therapy.

## Background

Measurement of invasive blood pressure (IBP) as reflection of blood flow and tissue perfusion is often carried out in animals, both during general anesthesia and in critical care.

Intravascular cannulation offers the potential to allow direct entry of gas into the circulation and can bring about the occurrence of arterial gas embolism. Indeed, in human medicine, arterial gas embolism can occur in two major situations: diving while breathing compressed air and during medical procedures such as vascular cannulation or intracavitary air insufflation [1, 2]. Cerebral air gas embolism (CAGE) may cause a spectrum of adverse effects ranging from very mild symptoms to severe neurological injury and death. In order to deepen the understanding of CAGE in human medicine, pigs, monkeys, rabbits, cats and dogs have been extensively used as models of air embolism [3]. However, to the best of the authors’ knowledge there are no reports of CAGE in calves and no reports of accidental iatrogenic CAGE from flushing of an arterial line in animals. The reported cases raise the concern that the injection of a small amount of air in a peripheral artery at high pressure, may lead to significant cerebral injury also in a clinical context. As the clinical presentation of CAGE is non-specific, misdiagnosis and delay of therapy are likely.

## Case presentation

A 7-day-old male Red Holstein–Friesian calf (No. 1) and a 28-day-old male Holstein–Friesian calf (No. 2) underwent respectively hot iron disbudding and sham disbudding in the context of a cross-controlled prospective clinical trial (ethical approval by Cantonal authority 2014_52_FR) investigating acute and chronic pain after disbudding. The procedure was standardized as following: after sedation with IM xylazine (0.1 mg/kg) an intravenous catheter was placed in a jugular vein and bilateral cornual nerve anesthesia (lidocaine 2%, 200 mg in total) was provided. In order to record physiologic nociceptive changes, heart rate (HR), respiratory rate (RR) and invasive blood pressure (IBP) were monitored during the procedure and hourly for the following 8 h. Prior to disbudding, an arterial cannula was placed in a caudal auricular artery and connected with the arterial monitor line previously filled with heparinized saline (100 IU/mL) from a fluid bag under 250 mmHg pressure. The bag was hanging vertically and only after verification that all parts were primed with fluids, the tubing system was connected to the arterial cannula. After zeroing the system at the height of the heart, to assess that the amount of damping was appropriate, the inline flushing device adjacent to the pressure transducer (Codan System DPT-6000, Codan Medical AG, Switzerland) was rapidly squeezed and released (fast flush test).

### Calf 1

**Fig. 1:**
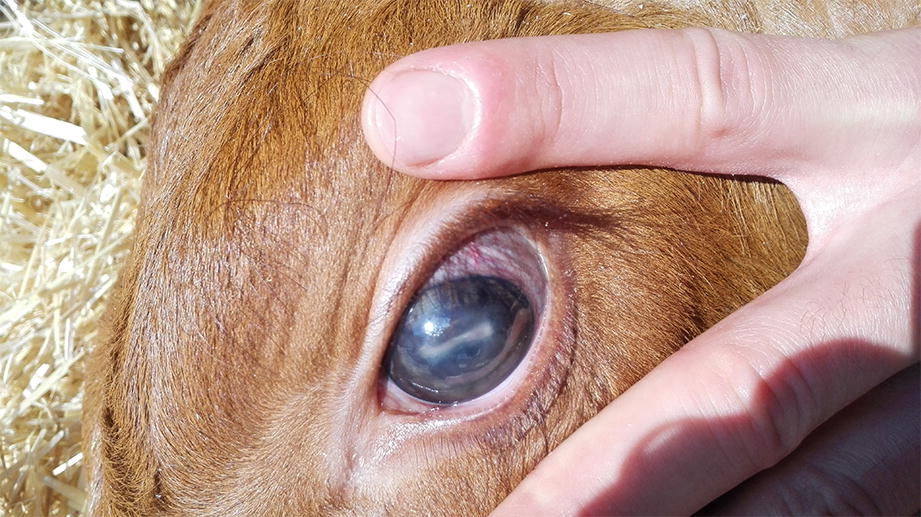
Glaucoma with opacity of the anterior chamber caused by acute, neutrophilic, fibrinous uveitis. Calf 1, left eye

Baseline IBP was 110/64/80 mmHg (SAP/DAP/MAP) and HR 79 beats per minute (bpm). Few seconds following the arterial flushing, IBP increased moderately (149/103/118 mmHg), and peak values were reached within 1 min (238/161/190 mmHg). During the hypertensive phase, HR first decreased slightly (68 bpm) and then increased up to 141 bpm. A short hyperpnoea (60 breaths per minute) was noticed. Concomitantly, the calf showed mild excitation, vocalization and purposeless movements, horizontal nystagmus and finally sensory depression. Over the following 10 min IBP decreased progressively to 166/126/141 and HR to 98. Disbudding was performed as foreseen. One hour later, sensory depression ameliorated and IBP returned to the baseline level. In the following 2 h a clinical improvement was observed, the calf could stand, yet it was reluctant to move. Four hours later, the calf could stand but right hemiparesis and severe ataxia were noticed and clear proprioceptive deficits on the right forelimb appeared. However, at that time, IBP, HR, RR remained in the baseline range. Neurologic examination revealed bilateral deficits of the V, VII and VIII cranial nerves. Sensory depression worsened progressively. An arterial blood sample taken 9 h after disbudding indicated mild hypoxemia (PaO_2_ = 78 mmHg with 0.2 FiO_2_), with normocapnia. Moderate hyperglycemia (6.6 mmol/L) was also present. When milk was offered, gag reflex was absent. Despite fluid and oxygen-supportive therapy during the night, the calf’ clinical conditions progressively worsened; the following morning, he was severely hemiparetic, stuporous and unable to drink. Blood gas and electrolyte analysis was unremarkable. Bilateral periocular edema and increased tension of the eye globe and opacity of the anterior chamber was noticed in the left eye (Fig. 1). Menace response was bilaterally absent. Euthanasia was performed for ethical reasons.

**Fig. 2:**
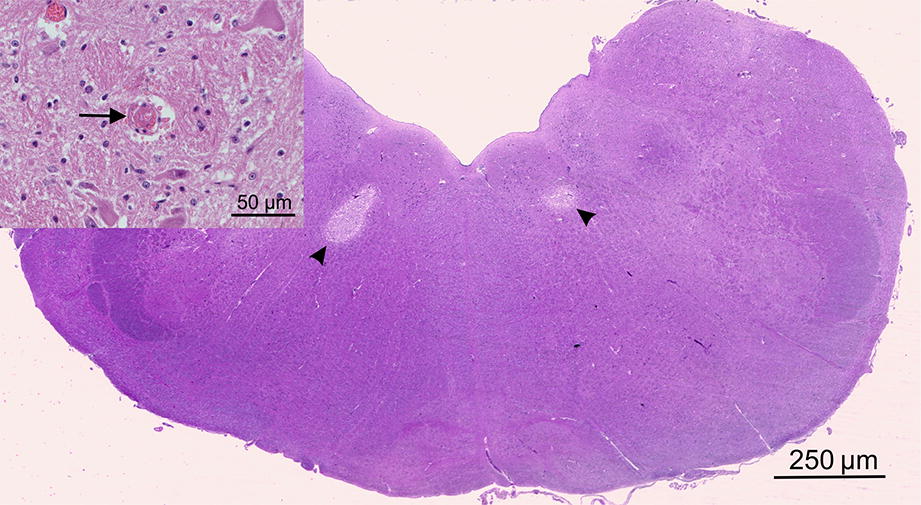
Photomicrograph showing two well demarcated infarcts, characterized decreased eosinophilic staining, with a bilateral and almost symmetrical distribution are present in the brainstem, ventrolaterally to the hypoglossal nuclei (arrowheads). Insert: In the vicinity of infarcts, capillaries are obstructed by fibrin thrombi (arrow) and surrounded by microhaemorrhages. Haematoxylin and eosin, brainstem, calf 2

Necropsy revealed a mild, diffuse, acute anterior uveitis with occlusion of the filtration angle and glaucoma in the left eye. Multifocal petechiae were noticed on the skin. In both lungs, a severe alveolar edema was present with neutrophilic infiltration of terminal bronchioli and surrounding alveoli, interpreted as a mild acute bronchopneumonia. Multifocal perivascular edema was noticed in the heart. The brain was macroscopically normal, but the histological examination revealed bilateral, well demarcated, infarct-like areas of necrosis in the brainstem, which were most conspicuous ventrolaterally to the hypoglossal nuclei (Fig. 2). These areas were characterized by edema, axonal swelling, neuronal eosinophilia and loosening of parenchyma with cavity formation. Small vessels within these areas had ill-defined and hypereosinophilic walls with necrotic endothelial cells (vessel wall necrosis) and contained degenerated neutrophils. Additionally, small infarcted areas were observed in the cerebellar vermis and in the cortex. Infarcts were associated with the presence of fibrin thrombi in small-sized vessels and capillaries and perivascular microhaemorrhages. These findings were interpreted as suggestive of disseminated intravascular coagulation (DIC).

Despite that, no entry of air bubbles into the arterial cannula was noticed; we suspected retrospectively a retrograde cerebral arterial embolism as air was noticed in the distal part of the tubing system.

### Calf 2

**Fig. 3:**
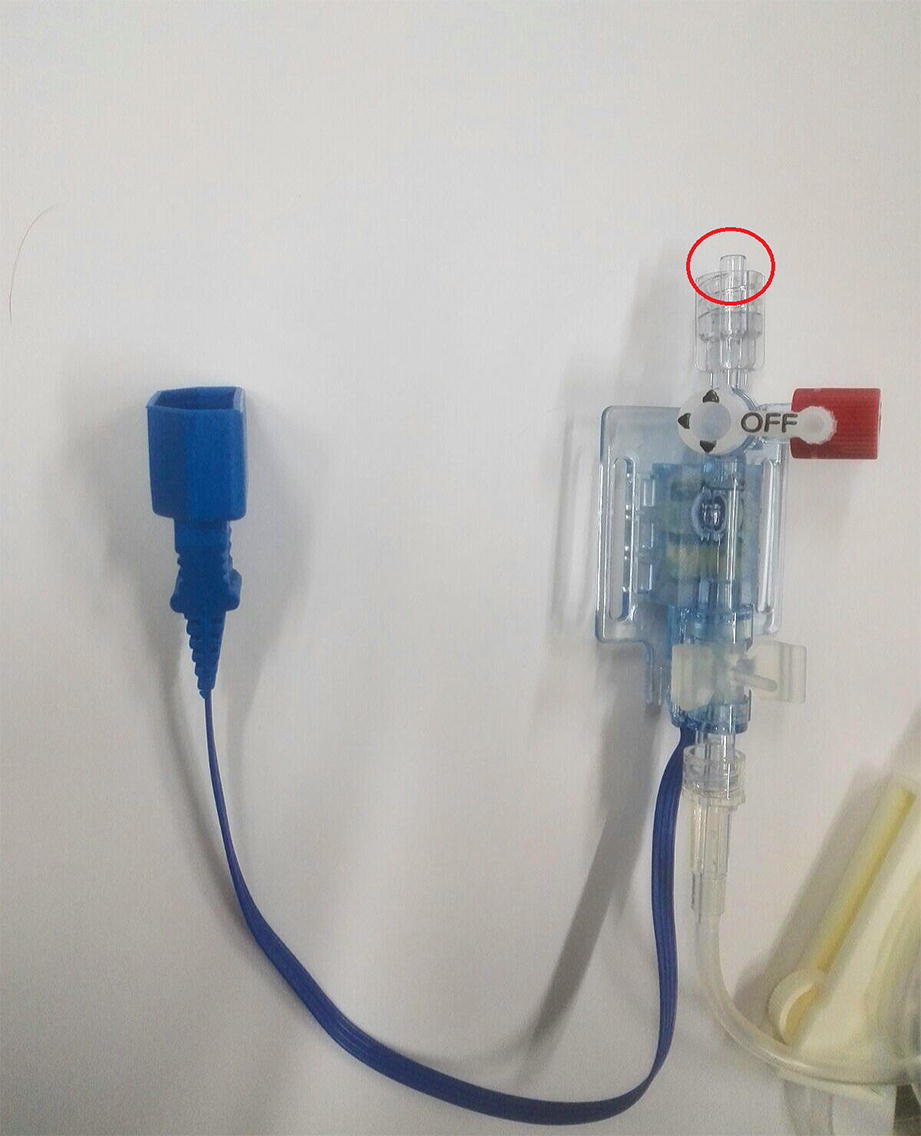
The untighten luer-lock in the blood pressure between the transducer indicated by a red circle

After successful placement of the arterial line, the tubing system was filled with the inline flushing device adjacent to the transducer and carefully checked for the presence of air bubbles. Baseline rectal temperature was 38.8 °C. IBP was 115/58/78 mmHg and HR 109 bpm. Thereafter, a fast-flush test was performed, and some bubbles were noticed entering the arterial cannula under high pressure. The investigators (DC and AM) recognized later on that there was a partial disconnection of the luer-lock connector between the flushing system and the transducer. We hypothesized therefore the same mechanism for calf 1 (Fig. 3). Few seconds after the inadvertent bubbles injection, the calf laid in lateral recumbency, showed excitation, hyperextension of the forelimbs, horizontal nystagmus and V and VII cranial nerve deficit. IBP pressure increased over 1 min up to 158/102/120 mmHg and concomitantly HR increased up to 128 bpm. The calf was repositioned in sternal recumbency, furosemide, 1 mg/kg, was injected IV and oxygen was provided. As the blood pressure increased further to 170/116/134, a second bolus of furosemide was injected (1 mg/kg IV). Over the next 2 min, IBP decreased to 153/92/112. HR decreased to 100 bpm and neurologic symptoms ceased. Sham disbudding was performed 1 min later when IBP was 138/74/95 mmHg and HR 98. One hour after disbudding, the calf was calm and still lying in sternal recumbency, but interactive. When encouraged, he could stand up without showing paresis. Blood pressure (115/60/80 mmHg) and HR (115 bpm) approached the baseline values. Slight hyperthermia (39.2 °C) was recorded. Respiratory rate remained in the normal range. No abnormalities were noticed in the following hours apart from slight hyperthermia (maximal peak 6 h later 39.6 °C). Behaviour and motor functions were deemed normal throughout the day; 8 h after disbudding, the calf showed normal temperature (38.8 °C) and normal appetite and it was reintroduced in the herd. The calf was observed for the following 3 months and it did not show either behavioral or neurological alterations.

## Discussion and conclusions

Gas embolism is defined as the entry of gas into vascular structures; arterial gas embolism can be caused by direct entry of gas into systemic arteries or indirectly by venous to arterial shunting [1, 2]. Arterial gas embolism is deemed rare in human medicine and occurs in two major situations: diving while breathing compressed air and direct entry of gas into the circulation during medical procedures such as vascular cannulation or intracavitary air insufflation [2, 3]. Arterial gas embolism causes ischemia in the organ in which the air bubbles are trapped. CAGE may cause a spectrum of adverse effects ranging from very mild symptoms to severe neurological injury and death. When bubbles occlude the brain vasculature, intracranial pressure increases and the distribution of blood flow in the brain becomes extremely inhomogeneous, leading to regional hyperemia and ischemia [4].

In order to facilitate advances in CAGE research, pigs, monkeys, rabbits, cats and dogs have been used in different models of air embolism [5]. However, to the best of the authors’ knowledge, there are no reports of CAGE in calves and no reports of accidental iatrogenic CAGE from flushing of arterial line in animals. On the contrary, retrograde cerebral air embolism is a recognized complication in humans after flushing of the radial arterial line [6–8]. In our case, the entry of air was due to partial disconnection of the tubing from the luer-lock; this report underlines the necessity of verifying that all the connections are secure, when setting an arterial line.

The symptoms of cerebral gas embolism develop suddenly, and it is worth underlying that there is no clear relationship between the volume of embolized gas and neurological symptoms [7–10]. Indeed, the clinical presentation shows marked variability favoring clinical CAGE misdiagnosis; in humans, symptoms may vary from minor motor weakness, dizziness, headache or moderate confusion to complete disorientation, hemiparesis, convulsions, anisocoria, loss of consciousness and coma [4].

When a bubble obstructs a cerebral vessel, three levels of interaction are present: the surface activity at blood gas-interface, the surface activity at endothelium interface and the obstruction per se leading to distal hypoxia and ischemia. Various plasma proteins including the coagulation system, complement and kinins are activated by bubbles. As a result, intravascular coagulation may be triggered and coagulopathies have been observed in animal models of cerebral embolism. The contact of the bubbles with the endothelium of the blood–brain barrier leads to development of an inflammatory response. Activation and adhesion of leucocytes have been implicated in the progressive fall of cerebral blood flow and decreased cerebral function. Brain swelling and inflammation may, therefore, play a stronger role in the occurrence of infarction by air emboli than they do in other forms of cerebral ischemia [4]. The interaction between ischemic and inflammatory process is dynamic and complex and may also explain why neuronal injury often extends beyond the area of arterial obstruction. Indeed, when only small amounts of arterial gas are embolized, insufficient to cause prolonged occlusion, there is a delayed reduction in cerebral blood flow even after the bubbles have dissipated [8]. A post-transit, gas-induced decrease in cerebral blood flow may explain why many patients who appear to have recovered from cerebral arterial gas embolism subsequently relapse. According to a rabbit model, this relapse should occur within 2 h after embolism [8]. In calf 1 the relapse was noticed within 4 h.

CAGE was diagnosed in the two calves according to the hyperacute appearance of the symptoms after an accidental injection of air into the arterial line, noticed in calf 2 and suspected in calf 1. The clinical suspicion of embolism is based on the initial neurologic symptoms and the direct temporal relation between these symptoms and the procedure [1, 4]. Histological findings in the brain of calf 1 are also strongly supportive of CAGE. In particular, the presence of ischemic infarcts, ischemic zones of cell loss, malacia, blanching of neurons and swelling of the perivascular spaces have been observed in dogs dying within 48 h after embolization. DIC was also observed in animal models of CAGE [4].

The severity of the symptoms was very different between the two calves, either in the early or in the delayed phase; although this is unsurprising according to the literature, it makes both a prompt diagnosis challenging and a prognosis difficult to be established. In our case, understanding modification of the sensory status associated with embolism was further complicated by the sedation caused by xylazine. In a group of dogs under anesthesia without muscle paralysis, respiratory abnormalities, extensory hypertonia and opisthotonus were observed as in our calves [9]. Arterial hypertension [9, 10], respiratory irregularities and bradycardia, consistent with increased intracranial pressure from a diffuse insult have been reported in experimental dogs [9], monkeys [6] and in human beings in clinical context [6] with various neurological sequelae. No permanent neurologic sequelae were observed in monkeys. In dogs recovering from anesthesia after air injection into the carotid artery, either no abnormalities or unilateral blindness, motor deficits ranging from mild hemiparesis to complete hemiplegia, involuntary movements and behavioral changes (depression or excitation) have been observed. Air embolism from radial arterial line culminated in brain death in a woman [6].

The fact that the animals were included in an experimental trial and that the facility hosting them was not designed for providing emergency treatment, limited our diagnostic and therapeutic choices. Advanced diagnostic imaging was not applicable to our animals. However, despite potentially indicative, neither computed tomography scan nor magnetic resonance imaging are considered conclusive in diagnosing CAGE [1]. In human medicine, initial treatment consists of administration of high concentration of oxygen to favor nitrogen wash out from the bubble into the alveoli, discontinuation of nitrous oxide administration if used during anesthesia and adequate patient positioning to maintain adequate blood pressure and open airway, and to prevent aspiration [3]. Both calves were positioned in sternal recumbency with head at the level of the heart; however, only calf 2 was supplemented with oxygen in the acute phase. Histologic exam of calf 1 revealed acute bronchopneumonia. We suspect that aspiration occurred when an attempt to feed him with milk was made. Although the level of evidence is not high, animal studies [11], case reports and retrospective studies on small numbers of patients suffering from cerebral arterial embolism have shown that hyperbaric oxygen therapy is the treatment of choice for this complication [3]. The use of hyperbaric oxygen therapy in veterinary patients is still in its early days and could not have been an option in our case either for practical or ethical reasons. We could have considered the use of intravenous lidocaine. Indeed, there are some evidences in experimental dogs [12] that lidocaine could be a useful adjunct to hyperbaric therapy due to its potential role in restoring the blood flow impaired by leucocyte adhesion and its efficacy to treat decompression illnesses in humans has been proven [13].

The inadvertent injection of air in the second calf happened after the death of the first calf. The presence of a massive pulmonary edema in the first calf was the main reason why the second calf was injected with furosemide. The other reason was the potential of furosemide for reducing brain water content. The authors are aware that for this purpose, the usefulness of furosemide has been proven only in combination with either mannitol or hypertonic saline [14–16], but unfortunately none of the two solutions was promptly available. Despite the fact that IBP stopped rising after the second injection of furosemide, it is difficult to distinguish whether this was dependent on furosemide or rather the phenomenon was self-limiting. Yet, the authors noticed that the stroke symptoms ceased more quickly than in calf 1.

Although subclinical, pulmonary edema in calf 1 deserves a further explanation. A non-cardiogenic pulmonary edema associated with severe brain injury is the most likely cause. Despite the pathophysiology is still debated, in experimental sheep, monkeys and dogs, an increase of intracranial pressure produced evidence of lung edema and in sheep high lung vascular permeability was demonstrated [17]. Pulmonary edema with or without hypoxia has been extensively associated with the administration of xylazine in sheep [18], but only hypoxemia has been observed in calves. The heart did not present any lesion which could explain a cardiogenic pulmonary edema.

In calf 2 we observed a slight hyperthermia which was self-limiting and it was not addressed. In humans, hyperthermia is a predictor of worse outcome in ischemic stroke, yet no information concerning its effect on CAGE is available [19]. In our case the peak of hyperthermia appeared after the resolution of neurological abnormalities; short term hyperthermia was also observed in several other calves in our study and potentially linked to xylazine [20, 21].

We could not exactly quantify the amount of air entering the arterial cannula, but being the arterial line already primed with fluids, the amount of air was likely tiny. Only some bubbles in a row were observed in calf 2. However, it seems that the rapid flushing plays an important role. A volume of air of 1.6 mL injected in the radial artery under high pressure was sufficient to evoke neurologic and hemodynamic reactions in an adult patient. It has been demonstrated in infants that rapid flushing not only results in retrograde embolization of flush solution, but also causes local arterial vasospasm, transient elevation of arterial blood pressure and intracranial/intraventricular pressure [22]. This can be related to the small size of the patient, but also to the immaturity of cardiovascular and nervous system.

The proximity of the arterial catheter to the cerebral circulation plays certainly a role. In children, the proximity to the carotid bifurcation is considered as a risk factor for intracranial lesions [22] and in adults, imaging of the cerebral circulation could be achieved with injection of fluorescein in a superficial temporal artery [23]. In the bovine species, the auricular artery is a branch of the external carotid artery. All blood destined for the circle of Willis first passes through the well-developed intracranial carotid rete. In bovines, the carotid rete, and hence the brain, receives most of its blood supply from branches of the internal maxillary artery, the continuation of the external carotid artery [24]. We can hypothesize that air injected into the auricular artery can consistently reach the brain: as several anastomoses are usually present between the left and the right rete, bilateral dispersion and damage after unilateral injection are very likely [24].

CAGE has never been reported in calves. The injection of a small amount of air in a peripheral artery in the vicinity of the cerebral circulation, at high pressure may lead to a significant cerebral injury. It is likely that iatrogenic arterial gas embolism occurs in the clinical veterinary context as well, but, due to the fact that clinical presentation shows marked variability, it could be either not recognized or misdiagnosed. As the consequences can be dramatic, special care must be taken performing the fast-flush test in arteries, especially if close to the brain.

## Abbreviations

bpm: beats per minute
CAGE: cerebral arterial gas embolism
DIC: disseminated intravascular coagulation
FiO_2_: inspired fraction of oxygen
HR: heart rate
IBP: invasive blood pressure
PaO_2_: arterial pressure of oxygen
RR: respiratory rate
